# Disinformation and Regime Survival

**DOI:** 10.1177/10659129241252811

**Published:** 2024-05-27

**Authors:** Yuko Sato, Felix Wiebrecht

**Affiliations:** 1 13148 Waseda University, Shinjuku-ku, Japan; 2 4591 University of Liverpool, Liverpool, UK

**Keywords:** disinformation, propaganda, democratization, autocratization, regime survival

## Abstract

Disinformation has transformed into a global issue and while it is seen as a growing concern to democracy today, autocrats have long used it as a part of their propaganda repertoire. Yet, no study has tested the effect of disinformation on regime stability and breakdown beyond country-specific studies. Drawing on novel measures from the Digital Society Project (DSP) estimating the levels of disinformation disseminated by governments across 148 countries between 2000–2022 and from the Episodes of Regime Transformation (ERT) dataset, we provide the first global comparative study of disinformation and survival of democratic and authoritarian regimes, respectively. The results show that in authoritarian regimes, disinformation helps rulers to stay in power as regimes with higher levels of disinformation are less likely to experience democratization episodes. In democracies, on the other hand, disinformation increases the probability of autocratization onsets. As such, this study is the first to provide comparative evidence on the negative effects of disinformation on democracy as well as on the prospects of democratization.

## Introduction

Disinformation by political actors is a growing concern worldwide, and its deleterious effects were becoming palpable even before the COVID-19 pandemic and Russia’s invasion of Ukraine (e.g., Bennett and Livingston 2018). Here, we concur with prior conceptualizations of disinformation and define it as purposefully created information that “has the function of misleading” (Fallis 2015, 422) and is “intentionally and verifiably false” (Allcott and Gentzkow 2017, 213). Although disinformation has long been part of dictatorships' propaganda machines, autocrats appear to have become more blatant in “spinning” false narratives in attempts to secure their hold on power (e.g., Guriev and Treisman 2022; Tenove 2020). Anti-pluralists and aspiring autocrats in democracies such as the United States, Brazil, Germany, and Sweden are also increasingly spreading “fake news” (e.g., Larsson 2020; Zimmermann and Kohring 2020). Targeted campaigns abroad by regimes such as Russia, China, and Iran are adding further stress to democracies (e.g., Hjorth and Adler-Nissen 2019; Pomerantsev 2015). As information is a fundamental resource for voters to hold governments accountable, disinformation is characterized as one of the key challenges to democracy (e.g., Benkler, Faris, and Roberts 2018).

Despite the abundance of disinformation and the growing concern around it, comparative research on the issue and its political consequences is rare (for exceptions see Piazza 2022, Humprecht, Esser, and Van Aelst 2020; Hunter 2023). In autocracies, disinformation is an inherent part of propaganda, but to date, empirical research has almost exclusively studied the cases of Russia and China (e.g., Huang 2015; Huang 2018; Rozenas and Stukal 2019). Here, the primary focus of these studies is on governments' strategies for information control and dissemination. At the same time, the macro-level political consequences, such as its effect on regime stability, have been understudied. In democracies, most prior research has focused on questions at the individual level, such as voters' exposure and susceptibility to disinformation (e.g., Enders et al. 2021; Erlich and Garner 2023; Hjorth and Adler-Nissen 2019), but also less on its consequences on the political system. Thus, it remains debated whether disinformation poses an immediate threat to democracy or remains a marginal phenomenon without far-reaching consequences (e.g., Allcott and Gentzkow 2017; Jungherr and Schroeder 2021; Lanoszka 2019).

In this paper, we seek to analyze the consequences of disinformation used by governments on political systems more specifically and combine insights from both regime types into a common framework of the effect of disinformation on regime survival. We argue that disinformation proves to be an effective tool for dictators to retain their hold on power and show that democratization is less likely in authoritarian regimes that disseminate more disinformation. On the other hand, we also echo concerns surrounding growing disinformation in democracies and suggest that higher levels of disinformation are associated with onsets of autocratization. Taken together, we suggest that disinformation is detrimental to democracy across regime types. In order to test this argument, we go beyond the existing China/Russia- (for autocracies) and US-centrism (for democracies) of prior studies and employ a comparative study. We draw on measures of disinformation from the Digital Society Project (DSP) (Mechkova et al. 2022) and combine it with the Episodes of Regime Transformation (ERT) dataset (Edgell et al. 2023) that identifies episodes of autocratization as well as liberalization. These datasets allow us to conduct a cross-national time-series study to systematically examine the effects of disinformation on regime survival across 148 countries between 2000 and 2022.

Empirically, we find that once we disaggregate the sample by regime types, our results show support for the regime-stabilizing function of disinformation in authoritarian regimes, making democratization episodes overall less likely. In democracies, higher levels of disinformation increase the probability of autocratization onsets and democratic breakdowns. To explain this finding, we point to the fact that disinformation in democracies promotes polarization in society, which inflates the risk of onsets of autocratization episodes. These results are robust to the inclusion of a number of control variables, alternative empirical modelings, and an instrumental variable (IV) analysis that addresses endogeneity concerns.

The contributions of this study are twofold. First, we overcome the scope limitations of previous studies and add further generalizability to prior studies on disinformation. Research on authoritarian regimes' use of disinformation is mostly based on findings from China and Russia (e.g., Huang 2015; Rozenas and Stukal 2019) while studies on disinformation’s threat to democracy primarily emanate from the United States (e.g., Tucker et al. 2018). As such, this study is the first to identify the link between disinformation and regime survival globally as well as across autocracies and democracies.

Second, the findings from this study contribute to debates about the impact of disinformation. Although research on authoritarian regimes has identified propaganda as a pillar of regime stability (e.g., Carter and Carter 2021; Huang 2015), others noted that propaganda and especially disinformation can also backfire (e.g., Huang 2018; Wang and Huang 2021). Adding to this debate, we find that across autocracies, disinformation can be an effective tool for dictators and is more likely to stabilize autocracies. Likewise, while many identify disinformation as a major challenge for democracies (e.g., Bennett and Livingston 2018), others see the reach of disinformation, and consequently, its influence as limited (e.g., Allcott and Gentzkow 2017; Grinberg et al. 2019; Guess, Nagler, and Tucker 2019; Lanoszka 2019). Despite such mixed results, we highlight that disinformation is associated with severe consequences, namely, the onset of autocratization episodes and democratic breakdown.

In the following, this paper will introduce its theoretical framework and hypotheses of how disinformation affects regime stability both in autocracies and democracies. Next, we introduce the empirical strategy to test these arguments. Then, we present the statistical results of the effect of disinformation on regime survival. Finally, we discuss the possible implications of our findings in the conclusion.

## Defining Disinformation

Especially since the presidency of Donald Trump, disinformation and “fake news” have received heightened attention in popular as well as academic discourses. While notions such as “misinformation,” “disinformation,” “conspiracy theory,” and “propaganda” are often used interchangeably, it is important to retain conceptual clarity. Starting from the broadest category, we follow prior research in this line of work and define misinformation as claims “that contradict or distort common understandings of verifiable facts” (Persily and Tucker 2020, 10). Disinformation, then, is a subcategory of misinformation with the difference being that it is purposely disseminated (e.g., Persily and Tucker 2020; Tucker et al. 2018). In other words, both concepts describe false claims but while misinformation may be shared accidentally and without malicious intent, disinformation “has the function of misleading” (Fallis 2015, 422) and deceiving. Although the question of intent may be difficult to prove, “organized attempts” of disseminating false information can be seen as a good indicator of such (Persily and Tucker 2020). Typical examples of disinformation may include Russia’s strategic disinformation campaigns domestically and abroad (e.g., Pomerantsev 2015) but also unsubstantiated claims of electoral fraud in the US (e.g., Berlinski et al. 2021).

As such, disinformation is different from other concepts such as conspiracy theories, even though they may often go hand in hand in practice. While truthfulness, or the lack thereof, is a defining element of disinformation, conspiracy theories, however, can rarely be classified as verifiably false and instead often share the belief into secretive elites that exercise control over society (e.g., Pirro and Taggart 2022; Sunstein and Vermeule 2009). Yet, disinformation shares some overlap with the concept of propaganda. The latter is defined as a “deliberate, systematic attempt to shape perceptions, manipulate cognitions, and direct behavior to achieve a response that furthers the desired intent of the propagandist” (Jowett and O’Donnell 2018, 6). As a broader strategy, this may and often does, contain disinformation (e.g., Lanoszka 2019) but also entails presenting and framing true pieces of information in a way that “disparages opposing viewpoints” (Tucker et al. 2018, 3). Elements of disinformation have therefore long been part of autocrats’ broader propaganda and information control strategies including in Rwanda and Nazi Germany (e.g., Adena et al. 2015; Yanagizawa-Drott 2014).

## Disinformation in Autocracies: Pillar of Regime Stability

Naturally, disinformation is more prevalent in dictatorships than in democracies (Boese et al. 2022). Not only do authoritarian regimes frequently disseminate disinformation but they also do so in an environment in which alternative channels of information are hardly available. In other words, disinformation from official channels is often the only ‘truth’ as it cannot be verified or triangulated with different sources (e.g., Gleditsch, Macías-Medellín, and Rivera 2023; Guriev and Treisman 2022). We hold that this strategy is an effective tool for dictators to remain in power as it hampers prospects for democratization in autocracies. In particular, it insulates dictators from mass protests, and we identify two primary ways in which disinformation affects citizens' willingness to protest.

First, disinformation can directly deflect responsibility and blame from dictators, making it more difficult for people to rally against them. In the absence of alternative sources of information and tight control of the internet, disinformation is significantly harder to detect, creating an opportunity for autocratic governments to present their performance better than it actually is (Boese et al. 2022). For example, authoritarian regimes regularly and frequently manipulate statistics on indicators such as economic growth (Martinez 2022) and deaths from COVID-19 (e.g., Annaka 2021). Instead, both Russia and China, for instance, repeatedly blame the West for bad economic situations and escalating tensions with them (e.g., Rozenas and Stukal 2019). Accordingly, disinformation often increases citizens' support for the regime while decreasing their motivation to protest against the government (e.g., Guriev and Treisman 2022). Even if citizens do not change their attitude about the government’s performance themselves as a result of propaganda, they may still believe that others have been persuaded, making coordination more difficult (e.g., Buckley et al. 2022; Huang and Cruz 2021). Consequently, in a regime that is dominated by propaganda and disinformation, consensus over the government’s performance is difficult to establish, and thus, collective action problems are unlikely to be solved. Ultimately, people may be less inclined to mobilize against the regime.

In addition, disinformation, similar to propaganda, may not only deter citizens from protesting because of its persuasiveness but may also signal the government’s strength to regime critics (e.g., Huang 2015). They may be in a better position to identify disinformation as such because of access to international sources and networks. Yet, blatant disinformation can showcase a regime’s grip on society and its intolerance for open debates. A regime that is willing to severely manipulate the information environment and disseminate false narratives, is likely to also resort to traditional forms of repression. For potential dissidents, extensive disinformation campaigns may signal the government’s extensive reach and make them less willing to protest in fear of likely repression. Indeed, prior work shows that digital repression including disinformation campaigns often goes hand in hand with traditional repression tools (e.g., Kendall-Taylor, Frantz, and Wright 2020). In consequence, as a government resorts more explicitly to disinformation, potential regime critics may abstain from protesting due to the fear of repression.

On the other hand, disinformation may not only disincentivize regime opponents from protesting against the regime, but it may also mobilize regime supporters to rally in favor of the government. Pieces of disinformation often claim to identify out-groups as perpetrators or causes of societal issues, whether they are internal (e.g., opposition actors, ethnic and religious minorities) or external (e.g., the US, the EU, or migrants generally). Simultaneously, this may enable autocrats to present themselves and their supporters as victims—a narrative that is often used to mobilize supporters (e.g., Ekiert, Perry, and Yan 2020; Pirro and Taggart 2022). Once pro-regime supporters are mobilized, these rallies also secure autocrats' hold on power since they signal strength and restrain mobilization against the regime (Hellmeier and Weidmann 2020).

In line with these suggested mechanisms, we hypothesize that high levels of disinformation are detrimental to the chances of democratization in authoritarian regimes. Our first hypothesis is thus, as follows:

H1: Autocracies are less likely to experience democratization when the government is more actively disseminating disinformation.

## Disinformation in Democracies: Threat of Autocratization

In addition to dictators' long-established propaganda strategies, political leaders in some democracies also increasingly use disinformation (Boese et al. 2022). In Poland, for example, public broadcasters had until 2023 increasingly become amplifiers of the previous right-wing government’s disinformation campaigns attacking migrants and discrediting civil society.^1^ Instances like this distort people’s preferences and pose a challenge to democratic systems' capacity for inclusion and reasoned deliberation (McKay and Tenove 2021). Therefore, we expect disinformation to be a significant factor in undermining democracies and making autocratization more likely.^2^

Disinformation is primarily disseminated by anti-pluralist parties and actors in government attempting to remain in power (Boese et al. 2022), including Republicans in the US (e.g., Allcott and Gentzkow 2017), radical right parties in Europe (e.g., Bennett and Livingston 2018; Hameleers and Minihold 2022), or pro-Russian parties in Ukraine (e.g., Erlich and Garner 2023; Peisakhin and Rozenas 2018). Through false stories targeting competitors (e.g., Pirro and Taggart 2022; Tenove 2020; Zimmermann and Kohring 2020), anti-pluralists attempt to boost their own popularity or avoid blame in government (e.g., Pirro and Taggart 2022). Unfortunately for democracy, being exposed to disinformation also has an impact on voting choices at least in some cases (Cantarella, Fraccaroli, and Volpe 2023; Zimmermann and Kohring 2020).

More generally, however, disinformation also threatens democracy due to its inherent potential to polarize society. Disinformation can affect citizens’ trust in democratic institutions and, thus, their preference for a democratic regime. Trump’s false allegations of electoral fraud in 2020, for instance, reduced trust in electoral integrity (e.g., Berlinski et al. 2021). Other examples include unjustified campaigns against expert commissions and institutions that undermine their credibility and trust in them (e.g., McKay and Tenove 2021). Often, an inherent part of disinformation is the use of “false claims, conspiracy theories, chauvinistic language, and visual imagery to stoke moral revulsion toward particular individuals, political parties, and social groups” (McKay and Tenove 2021, p. 709). In turn, disinformation and the branding of political opponents also inflate negative feelings and distrust, reinforce partisan identities, increase polarization of public opinion, and even instigate violence (Berlinski et al. 2021; Hjorth and Adler-Nissen 2019; Peisakhin and Rozenas 2018).

In such highly polarized contexts, voters may be more likely to sacrifice democratic principles to elect a candidate who champions their party or interests (Graham and Svolik 2020; Svolik 2019). Over time, these differences may transform into separate political camps with distinct understandings of what constitutes factual information. Such “pernicious polarization” (McCoy and Somer 2019) may be unsustainable for democracies.

Based on these proposed effects, we suggest that disinformation increases democracies' chances of autocratization and propose the following hypothesis:

H2: Democracies are more likely to experience autocratization when the government is more actively disseminating disinformation.

In sum, following these proposed theoretical mechanisms, we argue that disinformation stabilizes authoritarian regimes and destabilizes democracies. In the following, we will illustrate our research design and empirical strategy.

## Research Design

Despite burgeoning bodies of literature on disinformation and propaganda, comparative analyses are rare. Most existing work has focused on individual-level effects, primarily in the United States (for disinformation) as well as China and Russia (for propaganda). While these have led to tremendous progress in the study of these phenomena, we complement them with a comparative country-level study to understand their systemic effects on political regimes based on a global sample.

In order to test the relationship between disinformation and regime survival, we combine data from two primary sources. First, we utilize data from the Digital Society Project (DSP) (Mechkova et al. 2022). Among others, this data contains variables on online censorship, polarization, and politicization of social media from 2000 to 2022 around the globe.^3^ For the purposes of this paper, we are primarily interested in the variable that captures domestic disinformation efforts by the respective government (*v*2*smgovdom*).^4^ This is an expert-coded variable describing how prevalent the dissemination of false information by the government to influence its own population is and ranges from “extremely often” to “never or almost never.”^5^ The experts' ratings are aggregated using a Bayesian item response theory measurement model that can account for coder differences (Pemstein et al. 2018). This variable enables us to capture the core definition of disinformation—purposefully created information that “has the function of misleading” (Fallis 2015).

As a dependent variable, we consider two sets of variables. The first set of dependent variables is *onsets of democratization and autocratization episodes*. Democratization or autocratization episodes are defined as periods of substantial and sustained improvements or declines of democratic attributes measured with V-Dem’s Electoral Democracy Index (EDI) (Maerz et al. 2023). We call the first year of such episodes as democratization/autocratization “onset.” We utilize the Episodes of Regime Transformation (ERT) dataset (Edgell et al. 2023), which contains a complete sample of democratization and autocratization episodes between 1900 and 2022 that is comparable to existing datasets (Maerz et al. 2023).^6^ Here, we code the country-years in which a democratization or autocratization episode starts as one and zero otherwise. The country-years in ongoing episodes are excluded. We primarily estimate how the government’s use of disinformation increases or decreases the probability of a country experiencing the onset of a democratization or autocratization episode. However, we also consider how disinformation affects the likelihood of *democratic transition* and *democratic breakdown* as a result of democratization or autocratization episode onsets. Using the ERT dataset, we identify whether democratization or autocratization episodes resulted in a regime transition.^7^

**Figure 1.**
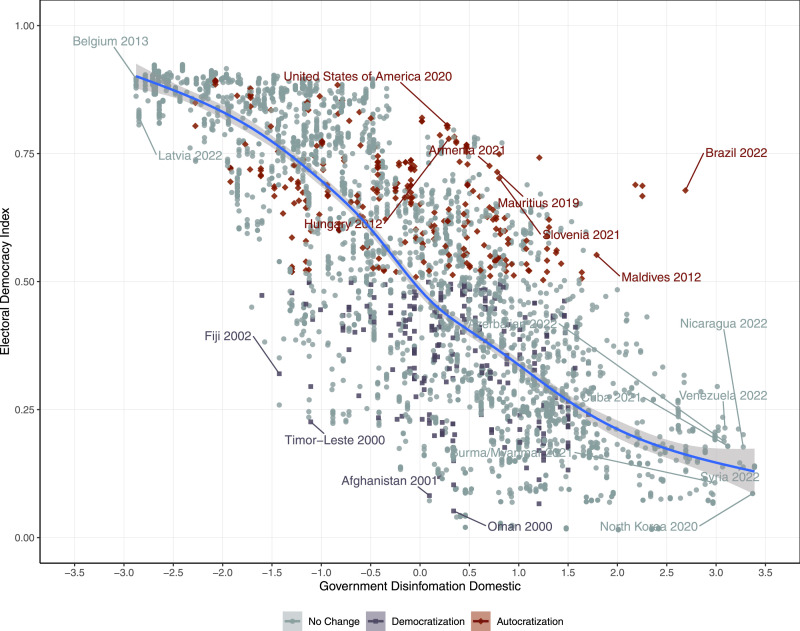
Correlation between Disinformation and the Level of Electoral Democracy. *Note*: Lines show the predicted levels of electoral democracy with 95% confidence intervals. Names of countries are labeled if the countries are in episodes of democratization or autocratization with the highest residuals or observed values of disinformation are less than the 1st percentile and higher than the 99th percentile of entire observations.

Figure 1 illustrates the general relationship between the level of disinformation and the EDI. The figure shows a clear negative relationship between the government’s use of disinformation and the quality of democracy. The figure shows that stable democracies (such as Belgium and Latvia) and autocracies (such as North Korea, Venezuela, and Cuba) are clustered at the lowest and highest end of the range of disinformation. Figure 1 further demonstrates the relationship between disinformation and whether countries democratized or autocratized in the observed year. Generally, countries undergoing autocratization tend to have higher levels of disinformation (above the fitted line), while states experiencing democratization tend to observe lower levels of disinformation (below the fitted line). In the cases of Fiji in 2002 and Afghanistan in 2001, the governments seem unable to effectively use disinformation despite their autocratic characteristics. On the other hand, while levels of democracy in Brazil in 2022 and the United States in 2020 are relatively high, the autocratizing government extensively uses disinformation. In sum, disinformation tends to correlate with both the levels of democracy and transition episodes. We further test these relationships using statistical models.

Second, to illustrate the mechanisms behind the relationship between disinformation and regime stability, we draw attention to disinformation’s potential for mobilization and polarization of society. First, as the theory indicates, the government’s use of disinformation, especially under autocracies, may decrease the citizens' willingness to protest against the government, while it is often used to mobilize the regime supporters to show their legitimacy. Second, disinformation under democracy tends to be used to polarize the voters intentionally. Therefore, we estimate the effect of disinformation on the levels of (i) mass mobilization and (ii) polarization of public opinion. Mass mobilization is measured by V-Dem’s mobilization for democracy (*v*2*cademmob*) and autocracy (*v*2*caautmob*) variables which measure the scale and frequency of pro-democratic and pro-autocratic mobilization in society. To measure polarization, we use V-Dem’s political polarization variable (*v*2*cacamps*), which captures the extent to which political differences affect social relationships beyond political discussions.^8^

### Empirical Approach

To estimate the relationship between the government’s use of disinformation and regime stability, we run two sets of statistical analyses: estimating the effects of disinformation on regime transformation and its mechanism. To demonstrate the heterogeneous effect of disinformation in different regime types, we further divide the sample into democratic and autocratic regimes based on V-Dem’s Regimes of the World (RoW) classification (Lührmann, Tannenberg, and Lindberg 2018). As our primary interest is the effect of disinformation on regime transitions, we exclude the analysis of autocratization in autocracies and democratization in democracies.

To estimate episode onset, we use a probit model in which those experiencing the beginning of an episode are treated as ones. Since we use panel data, we have taken several measures to account for different forms of estimation error. First, we use clustered standard errors by countries to account for heteroskedasticity as well as autocorrelation. Second, we employ fixed-effects models to account for unobservable confounding effects derived from country- or year-specific characteristics. Here, however, we encounter a common issue in fixed-effects analyses of dichotomous dependent variables that capture relatively rare events: the separation problem, in which dropping non-event-experiencing units from the analysis results in bias. To address this issue, we first relax some assumptions and include regional dummies and a linear time trend to account for global trends instead of country- and year-fixed effects. In addition, we present both conditional probit and linear versions of our fixed-effects specifications, with the latter making use of all observations. We follow the recommendations of Beck (2020) in reporting marginal effects for our key independent variables from fixed-effects probit analyses and linear fixed-effects analyses using the same limited sample to allow for a meaningful comparison between the specifications.^9^

Second, to estimate the relationship between disinformation and polarization and mobilization, we use OLS models with country- and year-fixed effects taking account of all unobservable factors.

To estimate the relationships, we include a number of control variables to account for potential confounding factors between disinformation and regime survival. The first set of variables relates to countries' economic situation. Since Lipset’s (1959) seminal work, existing studies indicate a strong correlation between levels of economic development and democratic regimes' development and stability (e.g., Przeworski and Limongi 1997). In addition, studies indicate that negative economic growth is a predictor of regime breakdown either through democratization (e.g., Teorell 2010) or democratic breakdown (e.g., Bernhard, Reenock, and Nordstrom 2004). Such economic conditions may also affect the government’s capacity to use disinformation (Rozenas and Stukal 2019). Thus, we control for *GDP* per capita and *GDP growth rate*, extracted from the World Bank (WDI 2023). As autocratic leaders often use education as a tool of indoctrination for their ideology, such levels of indoctrination affect both the voters' susceptibility to the government’s disinformation efforts and their support for the regime. Accordingly, we control for *indoctrination potential in education* (*v*2*xed*_*ed*_*inpt*) from the V-Dem dataset (Coppedge et al. 2023; Northmore-Ball et al. 2023).

The second set of variables relates to the government’s motivation to use a disinformation strategy. First, we control for *the population size* as it might affect a polity’s susceptibility to disinformation and regime change. Second, we control for *internet penetration rates*. As contemporary disinformation primarily spreads through the internet and social media (e.g., Trauthig, Martin, and Woolley 2023), the effect of disinformation on democratization or autocratization may depend on the share of internet users among the population. Third, we control for *the government’s internet filtering capacity* to account for the extent to which a government can produce and disseminate disinformation in the first place. This variable is also taken from the DSP.

The last set of variables relates to countries' democratic embeddedness. First, we include *the regional levels of democracy* across six world regions in the empirical models. This variable controls for the diffusion effects of democratization (e.g., Brinks and Coppedge 2006) and autocratization (e.g., Lührmann and Lindberg 2019) from neighboring countries and the government’s motivation of controlling information during such episodes. Second, we control for countries' previous experience under democracy (*democratic stock*). Studies indicate that the institutionalization of democratic institutions increases the probability of democratization and the stability of democracy (Boese et al. 2021; Svolik 2015). We draw on a recently developed measure of democratic stock based on V-Dem’s EDI (Edgell et al. 2020).

Descriptive statistics of all variables used in our empirical models are presented in Table A1 in the Appendix.

### Potential Endogeneity and Instrument

Regime transformation is associated with technological change, that is, the liberalization of information and communications technology increases the probability of democratization, or dictators limit the diffusion of information to lengthen their time in office (e.g., Knutsen 2015). At the same time, leaders only choose disinformation strategies to avert democratization (autocracies) or promote autocratization (democracies) if the internet is highly influential in citizens' lives. Accordingly, our model estimates are likely biased if we include internet penetration due to the potential confounding effects. The exclusion of internet penetration from the model will also cause the estimate to be biased since, in that case, disinformation may capture the impact of the internet on regime transformation through other ways than disinformation.

In addition, recent studies increasingly reveal the shortcomings of fixed effects regressions to show the causal effect with the longitudinal data (e.g., Imai and Kim 2019; Imai and Kim 2021). The models are fundamentally based on the within-unit comparison, and they presume the absence of dependency between the past outcome and the treatment assignment, which is potentially violated in this study.

To alleviate such endogeneity concerns, we perform an instrumental variable (IV) analysis. Following Jha and Kodila-Tedika (2020), we use technological adoption in communication in 1500 CE as an instrument for internet penetration. As Comin, Easterly, and Gong (2010) show, cross-country differences in technological adoption in the communication industry in 1500 CE can explain current cross-country variations in technological states. This is because the technological advantage, such as lower cost of adopting new technology and innovation, economies of scale, and cross-sectoral technological spillovers, persists over a long time. The data for the technological adoption index in communication in 1500 CE is extracted from the Cross-country Historical Adoption of Technology (CHAT) dataset (Comin and Hobijn 2009). In addition, to incorporate the global levels of technological advancement over time, we use the exogenous variation in average worldwide internet penetration following Hunter (2023).

Thus, we consider the instrument as the interaction of a state-specific variable (technological adoption in communication in 1500 CE) and the time-invariant variable (average worldwide internet penetration).

Since there is little reason to believe that technological adoption in communication in 1500 CE and the average worldwide internet penetration rate will affect the likelihood of regime transformation today other than via its effects on internet penetration, they are valid instruments. Moreover, we find that the technological adoption in communication in 1500 CE, as well as the average worldwide internet penetration, are strong predictors of internet penetration today, making them robust instruments.

## Results

First, we disaggregate the sample into democratic and autocratic regimes (Table 1) and show if the level of disinformation affects episode onsets. Models 1–3 indicate the effects of variables on the probability of democratization onset in autocracies. The results demonstrate that the government’s use of disinformation negatively affects the probability of democratization onset, and the effect is statistically significant at high confidence levels. The results hold when including country- and year-fixed effects and the marginal effect of the probit model is comparable to that of the linear probability model (Table B1 in Appendix). In addition, we present a baseline treatment effect without any covariates to alleviate the potential overfitting problem of including too many controls in a two-way fixed-effects model (Table B2 in Appendix). By excluding all covariates, we found a consistent result with the main estimate.

**Table 1.** The Effect of Disinformation on Democratization and Autocratization Onsets.

	Dependent variables:					
	(1)	(2)	(3)	(4)	(5)	(6)
	Democratization onset			Autocratization onset		
Disinformation	−0.322***	−0.519***	−0.039**	0.285***	0.731***	0.070**
	(0.065)	(0.085)	(0.018)	(0.077)	(0.116)	(0.029)
Internet penetration	−0.580	−1.005 ***	−0.063	0.399	2.088***	−0.032
	(0.469)	(0.375)	(0.062)	(0.546)	(0.487)	(0.080)
Indoctrination potential	−0.446*	−0.322	−0.020	0.244	0.852	0.155
	(0.251)	(0.370)	(0.087)	(0.406)	(0.747)	(0.215)
GDP growth	−0.028***	−0.032***	−0.003*	−0.018*	−0.011	−0.002
	(0.010)	(0.006)	(0.002)	(0.010)	(0.007)	(0.001)
GDP per capita (log)	−0.051	0.109	−0.004	−0.224**	−0.486***	−0.005
	(0.073)	(0.094)	(0.015)	(0.103)	(0.161)	(0.026)
Population (log)	−0.064*	−1.687***	−0.114*	0.073	3.685***	0.191*
	(0.037)	(0.384)	(0.066)	(0.049)	(0.733)	(0.100)
Internet filtering capacity	0.029	−0.137	−0.013	0.301***	0.309***	0.030*
	(0.055)	(0.119)	(0.020)	(0.064)	(0.089)	(0.018)
Democratic stock	−0.058	−19.550***	−1.955**	1.530	68.822***	1.643
	(0.982)	(4.615)	(0.787)	(0.931)	(5.833)	(0.698)
Regional democracy levels		2.834	0.220		20.451***	0.617
		(2.382)	(0.481)		(2.565)	(0.410)
*t*	0.060			0.121***		
	(0.040)			(0.039)		
*t* ^*2*^	−0.002			−0.005***		
	(0.002)			(0.002)		
Constant	−0.099	26.309***	2.010*	−1.434	−99.710***	−4.203**
	(0.858)	(6.488)	(1.135)	(0.988)	(13.161)	(1.844)
Sample	Autocracies			Democracies		
Observations	1314	1311	1311	1454	1452	1452
Log likelihood	−150.368	−127.990		−143.230	−107.654	
Adjusted *R*^2^			0.110			0.077
Region FE	YES	NO	NO	YES	NO	NO
Country FE	NO	YES	YES	NO	YES	YES
Year FE	NO	YES	YES	NO	YES	YES
Model	Probit	Probit	OLS	Probit	Probit	OLS

*Notes*: Standard errors clustered by country. ***, **, * significant at 0.01, 0.05, 0.10, respectively. Countries that do not have multiple-year observations are excluded from the analysis for two-way fixed-effects model (Models 2, 3, 5, and 6).

Next, Models 4–6 indicate the effect of variables on the likelihood of an autocratization onset in democracies. The effect of government disinformation is positive and statistically significant indicating that disinformation increases the probability of a country to start autocratizing. This result is consistent when including country- and year-fixed effects (Models 5 and 6, and Table B1 in Appendix) and excluding all covariates (Table B2 in Appendix). The results indicate that disinformation decreases the stability of democracies.

**Figure 2.**
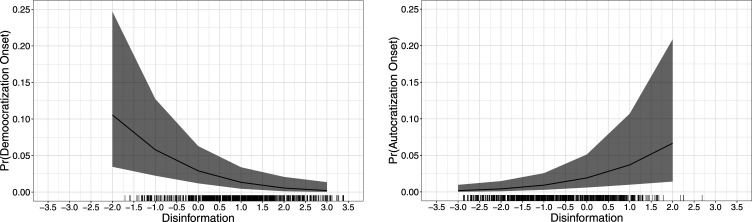
Predicted probabilities (democratization and autocratization onsets). *Note*: Lines show the predicted probabilities with the 95% confidence intervals. The left figure is generated from Model 1 and the right figure is generated from Model 4 of Table 1.

Figure 2 further shows the predicted probabilities of democratization onsets in autocracies (left panel) and autocratization onsets in democracies (right panel). The left panel indicates that the predicted probabilities of democratization onset decrease on average from 10% to 0% when the disinformation scores move from the lowest to the highest in autocracies. On the other hand, the right panel indicates that the predicted probability of an autocratization onset increases as governments' use of disinformation increases. This increase amounts to, on average, from 0% to 7% when disinformation levels move from the lowest to the highest in democracies.

Next, we test how disinformation strategies used by governments affect regime stability using the two-step model developed by Boese et al. (2021). In this analysis, we estimate both the probability of (i) the onset of episodes and (ii) the breakdown of the regime (democratic transition or breakdown) separately. Models 11–12 in Table B3 in Appendix test the effects of variables on selection into democratization episodes and democratic transition. The results indicate that the government’s disinformation strategy significantly decreases the probability of democratization onsets. Model 12 assessing the effects of factors on democratic transition also indicates that disinformation has negative and statistically significant effects. Thus, the result demonstrates that disinformation is effective in preventing democratization onsets and democratic transition altogether.

Models 13–14 in Table B3 indicate the effects of variables on selection into autocratization episodes and democratic breakdown, respectively. Model 13 indicates that the government’s use of disinformation significantly increases the probability that the country will experience the onset of autocratization. The coefficient of the second stage is also positive and statistically significant, indicating that it affects the probability of democratic breakdown.

Thus, we find empirical support for the two hypotheses: (i) autocracies are less likely to experience democratization when high levels of disinformation are prevalent (H1) and (ii) democracies are more likely to experience autocratization when high levels of disinformation are prevalent (H2). In addition, we find that in both autocratic and democratic regimes, the government’s disinformation strategy is significantly associated with both episodes' onsets and (the absence of) regime transitions.

### Sensitivity Test

Next, we present the results from the models using an instrumental variable approach (Table 2). We instrument internet penetration with the technological adoption in communication in 1500 CE and average global internet penetration. The technological adoption in communication in 1500 and global internet penetration rates jointly are significant predictors of internet penetration in both models in autocracies and democracies. Specifically, the F-statistics is greater than the rule-of-thumb value of 10, suggesting that the instruments are strong. With this specification, the effects of disinformation are compatible with the main models in Table 1. Moreover, the coefficient for internet penetration for both models is not statistically significant, suggesting that disinformation is the mechanism through which the internet promotes or averts regime transformation. The results add confidence to our main results.^10^

**Table 2.** Instrument Variable Approach.

	Dependent variable:	
	Democratization	Autocratization
	(7)	(8)
First-stage regression. DV: Internet penetration		
Comm.tech. in 1500 CE	−0.491***	0.675***
	(0.149)	(0.086)
Ave. Internet penetration	0.922***	0.714***
	(0.071)	(0.057)
Comm.tech. in 1500 CE*Ave. Internet penetration	0.307***	−0.242***
	(0.070)	(0.032)
F statistics	18.980***	57.334***
Second-stage regression. DV: Democratization/Autocratization		
**Disinformation**	−0.055**	0.054*
	(0.027)	(0.032)
Internet penetration (predicted)	0.173	−0.010
	(0.438)	(0.362)
Indoctrination potential	0.002	0.244
	(0.143)	(0.285)
GDP growth	−0.002	−0.002
	(0.002)	(0.002)
GDP per capita (log)	−0.019	0.029
	(0.020)	(0.027)
Population (log)	−0.055	0.177
	(0.200)	(0.135)
Internet filtering capacity	−0.005	0.035
	(0.028)	(0.024)
Democratic stock	−1.113	1.210
	(1.959)	(1.138)
Regional democracy levels	0.500	0.814
	(0.903)	(1.205)
Constant	0.916	−4.252
	(3.676)	(2.735)

Sample	Autocracies	Democracies
Observations	820	1114
Adjusted *R*^2^	0.008	0.088
Country FE	YES	YES
Year FE	YES	YES

*Notes*: Standard errors clustered by country. ***, **, * significant at 0.01, 0.05, 0.10, respectively.

### Mechanisms

In order to illustrate the mechanisms behind the findings above, we draw attention to disinformation’s potential for mobilization and polarization of society. We test how disinformation affects regime stability through increasing/declining polarization and mobilization of society (Table 3). The models indicate the effects of disinformation on *Mass Mobilization for Democracy* (Models 9–10), *Mass Mobilization for Autocracy* (Models 11–12), and *Political Polarization* (Models 13–14).

**Table 3.** Mechanisms.

	Dependent variable					
		Dem. Mobilization		Aut. Mobilization		Polarization	
Sample	Aut.	Dem.	Aut.	Dem.	Aut.	Dem.
	(9)	(10)	(11)	(12)	(13)	(14)
**Disinformation**	0.086	0.375^***^	0.384^***^	0.308^***^	0.460^***^	0.424^***^
	(0.132)	(0.102)	(0.121)	(0.117)	(0.105)	(0.065)
Internet penetration	*−*0.176	*−*0.528^**^	0.257	*−*0.193	0.235	*−*0.384
	(0.308)	(0.256)	(0.322)	(0.258)	(0.324)	(0.236)
Indoctrination potential	*−*1.269	1.787^*^	0.127	0.850	0.029	1.637^**^
	(0.827)	(0.974)	(0.434)	(0.733)	(0.366)	(0.752)
GDP growth	*−*0.016^**^	0.006	*−*0.007^*^	0.005	*−*0.005^*^	0.001
	(0.007)	(0.005)	(0.004)	(0.005)	(0.003)	(0.004)
GDP per capita (log)	*−*0.390^**^	0.009	0.094	*−*0.008	*−*0.221^***^	*−*0.147
	(0.192)	(0.116)	(0.073)	(0.074)	(0.069)	(0.097)
Population (log)	*−*0.549	*−*1.357^***^	*−*0.090	*−*0.758^*^	*−*0.105	*−*0.722^*^
	(0.353)	(0.366)	(0.289)	(0.392)	(0.345)	(0.385)
Internet filtering capacity	0.003	*−*0.075	0.029	*−*0.123	0.076	*−*0.144^*^
	(0.100)	(0.081)	(0.098)	(0.083)	(0.104)	(0.077)
Democratic stock	0.473	2.907	10.728^**^	2.087	2.773	1.805
	(6.474)	(3.561)	(4.579)	(3.437)	(5.635)	(3.263)
Regional democracy levels	*−*2.195	0.905	*−*0.673	0.604	*−*0.914	*−*2.131
	(1.863)	(1.294)	(1.703)	(1.546)	(1.753)	(1.728)
Constant	12.266^*^	22.957^***^	0.233	11.469	2.899	15.576^**^
	(6.619)	(6.616)	(4.932)	(7.068)	(5.674)	(7.057)
Country FE	YES	YES	YES	YES	YES	YES
Year FE	YES	YES	YES	YES	YES	YES
Observations	1462	1636	1462	1645	1462	1655
Adjusted *R*^2^	0.771	0.843	0.850	0.880	0.872	0.928

*Notes*: Standard errors clustered by country. ***, **, * significant at 0.01, 0.05, 0.10, respectively.

In authoritarian regimes, while disinformation positively affects the levels of polarization (Model 13), the government’s use of disinformation does not promote pro-democratic mobilization (Model 9). On the other hand, disinformation increases the level of autocratic mobilization (Model 11). However, using the instrumental variable approach (Table C1 in Appendix), we find that disinformation does not increase either form of mobilization or polarization in autocracies. These results may indicate that the observed effects of disinformation are due to the confounding effects of internet penetration, e.g., internet availability increases both the probability of democratization and the government’s use of disinformation. Nevertheless, this result is in line with the findings that disinformation helps dictators avoid democratization and keep the status quo. Regimes with the highest levels of disinformation generally avoid liberalization episodes altogether.

Second, in democracies, the government’s use of disinformation has a strongly positive and significant impact on political polarization (Model 14), meaning higher levels of disinformation are linked to a more polarized society. Such political polarization may destabilize democracy and thus create an opportunity for autocratization onsets. In line with this, the results from Models 10 and 12 further indicate that the government’s disinformation increases both pro-democratic and pro-autocratic mobilization in democracies. These results are consistent when using the instrumental variable approach (Table C1), which adds confidence to our findings. Thus, disinformation in democracies can destabilize society and may lead to an onset of autocratization.

In sum, we find strong evidence that disinformation helps dictators to remain in power and makes democratization less likely. In democracies, since democracies retain alternative sources of accurate information, disinformation will rather polarize society. Such polarization may create an opportunity for democratic breakdown.

## Robustness Check

We ran several robustness tests to check the sensitivity of our results. We conduct robustness checks on the effect of disinformation by considering alternative datasets and model specifications.

First, our estimate may be sensitive to the identification of regime transformation episodes, which are determined by the ERT dataset (Edgell et al. 2023) (i.e., +/− 0.10 overall change of the EDI). Table B4 shows the effect of disinformation on the probability of regime transformation onsets is consistent with the main result (Table 1) using either a lower (+/− 0.075) or higher (+/− 0.125) threshold.

Second, testing the sensitivity of the findings is especially important for estimating the probabilities of a rare event. The models in Table B5 of the Appendix show the results of the duration models (Beck, Katz, and Tucker 1998). The effect of disinformation on democratization onsets is consistent with the main result, namely that disinformation decreases the probability of democratization in autocracies. The effect of disinformation on autocratization onsets in democracies, however, disappears once we control for autocorrelation effects, while the direction of the effect is consistent with the main result. This null finding may be because of the limited time period (21 years) combined with the rare events^11^ that make it difficult to estimate the relationship with the duration model. Using the residual as a dependent variable to alleviate problems in estimation using a rare event as a dependent variable (McGrath 2015) (Table B6); however, we found all consistent results with the main models in Table 1.

Third, we present analyses with different lags of the independent variables (Table B7 in Appendix). The result indicates that, in autocracies, disinformation has a consistent and negative effect on democratization onsets between *t* to *t−* 4. However, when we use the change in the level of disinformation between the observation year and the year before to account for the possible autocorrelation effect (Table B8), the result is still consistent with the main models in Table 1. On the other hand, in democracies, only disinformation at *t* and *t −* 5 have significant effects promoting autocratization onset. The result may indicate that disinformation has relatively short-term effects on autocratization onsets. Table B8 also shows that the change in disinformation strongly and significantly affects autocratization onset.

Next, we include additional control variables that may also affect the level of democracy and probabilities of democratization or autocratization onsets: state capacity taken from Hanson and Sigman (2021) (Table B9), state repression capacity extracted from Fariss and Schnakenberg (2014) (Table B10), government’s control over social media (Table B11), judicial and legislative constraints on the executive (Table B12) both taken from the V-Dem’s dataset (Coppedge et al. 2023), and the occurrence of coups in observation years taken from Albrecht, Koehler, and Schutz (2021) (Table B13). The results are largely consistent with the main results, adding confidence to our findings.

In addition, we test if the general relationship holds by excluding the commonly studied cases where disinformation is used, Russia, China, and the United States (Table B14). By excluding these cases, disinformation still affects the probability of democratization and autocratization onsets. Thus, the statistical result indicates that the observed relationship is not only driven by the commonly studied cases but equally applies to a broad sample.

Finally, we show a robustness test for the mechanisms (Table 3). Here, we use alternative sources of data to estimate both pro-democratic mobilization and polarization.^12^ Models 7–8 in Table C2 show the effects of variables on the number of pro-democratic mobilizations observed in the specific country-year. The data is extracted from the Mass Mobilization Data (Clark and Regan 2018). In addition, we replicate the analysis using the mass affective polarization score proposed by Wagner (2021) using the Comparative Study of Electoral Systems (CSES) dataset (Model 9 in Table C2).^13^ Using these alternative datasets, we found consistent results with the main analysis.

In sum, we find the most consistent and robust empirical support for Hypothesis 1 – autocracies are less likely to experience democratization when high levels of disinformation are prevalent. The positive effect of disinformation on autocratization onset in democracies (Hypothesis 2) also holds across different model specifications.

## Conclusion

Disinformation is a growing concern worldwide especially given that it seems to coincide with the current wave of autocratizaton (e.g., Wiebrecht et al. 2023). While disinformation campaigns have been a prominent tool for autocracies as part of their propaganda and information control schemes, such strategies are also increasingly used by anti-pluralist leaders in democracies and are widely seen as a threat to democracy. Yet, despite the importance of the issue, cross-national evidence systematically demonstrating the effect of disinformation on regime stability has been largely absent. This study is the first to fill this gap by conducting cross-national time-series analyses with a global sample, testing the effect of disinformation on both democratic and autocratic stability.

Our findings provide robust evidence for the fact that disinformation negatively affects the quality of democracy in any regime type and highlight that disinformation helps dictators to remain in power as it reduces the likelihood of democratization in autocracies. We also find that disinformation is linked to the onset of autocratization episodes as well as democratic breakdown. These findings are robust to most model specifications, including an instrumental variable approach tackling the endogeneity problem. We trace these results back to the mechanism that disinformation in autocracies helps the status quo preventing pro-democratic mobilization from emerging. On the other hand, in democracies, disinformation may not necessarily be the leading cause of autocratizaton but we show that it leads to higher levels of societal polarization into separate camps of those who believe in false information (supporting the government) and those who do not (opposing the government). This polarization, in which both pro- and anti-democratic forces mobilize more intensely, can create an opportunity for autocratization as we see from recent cases such as the United States, Brazil, Poland, and Hungary.

These findings have important implications. First, our study reveals the effectiveness of the disinformation strategy used in autocracies to keep their regimes stable. Future research may test the different mechanisms more explicitly, especially in contexts other than Russia and China to expand our knowledge of disinformation in authoritarian regimes. Second, we confirm the warnings of disinformation as a threat to democracy empirically and therefore echo calls that countering disinformation becomes increasingly important (Winger et al. 2023). On the other hand, our results also suggest a backlash against disinformation from civil society and pro-democracy actors, which can be critical for resisting autocratization (e.g., Bowler, Carreras, and Merolla 2023; Tomini, Gibril, and Bochev 2023).

## Supplemental Material

Supplemental Material - Disinformation and Regime Survival Supplemental Material for Disinformation and Regime Survival by Yuko Sato and Felix Wiebrecht in Political Research Quarterly.

## Notes

1. https://ipi.media/polands-division-hinders-fight-against-fake-news-2/.

2. However, we also acknowledge that isolating the effect of disinformation is nearly impossible since it is often used by actors that advance autocratization simultaneously in several areas. Therefore, disinformation may not necessarily be the leading cause of autocratization, but as we show later in the paper, it appears to have a causal effect on increased polarization in society which is also a significant factor for autocratization (McCoy, Rahman, and Somer 2018).

3. Despite a limited time scope due to data availability, our analysis covers the most relevant period for evaluating our hypotheses as internet and social media accessibility significantly increased during this period.

4. It is important to note here that we study disinformation disseminated by the government in their respective countries. We do not study disinformation campaigns targeting other countries, such as Russia’s efforts in Central and Eastern Europe or China’s meddling in Taiwan. These cases go beyond the scope of this paper.

5. For presentation purposes, we reverse the order of the responses so that “never, or almost never” becomes the lowest score and “extremely often” the highest score. In addition, we primarily look at the *level* of government use of disinformation, but we also test the effect of *change* in the level of disinformation as a robustness check to take into account the temporal dependency in the variable.

6. See Appendix and Maerz et al. (2023) for more details. Table A2 of the Appendix summarizes democratization and autocratization episodes included in the models. We also present robustness tests using alternative thresholds to identify these regime transformation episodes (Table B4 in Appendix).

7. It may be worth mentioning that although our dependent and independent variables can both be found in the V-Dem dataset, they are part of different expert surveys and are therefore, not subject to a bias in which the same experts code both of our variables. A skeptical reader may suggest that coders may be more familiar with cases of regime changes and might have used that as a heuristic to assess the levels of disinformation in the respective country. However, as Weidmann (2023) recently shows, there is very little evidence of an availability bias, that is, recent events in the country in question driving coder decisions of rating.

8. Again, it may be worth making explicit that these variables are generated through different expert surveys than our key independent variable. In addition, we run a robustness check with alternative data sources (Table C2 in Appendix).

9. To illustrate the main results, we primarily rely on the first model with regional dummies. Estimation with a two-way fixed-effects model in this study can be critically vulnerable by dropping off 56 countries or 869 observations in autocracies and 58 countries or 1033 observations in democracies. Differences in samples may alter the estimates, especially if the size of the omitted group is large (Beck 2020).

10. We note that this result may need to be cautiously understood. By applying the instrumental variable approach, the models drop some observations due to the missing data on technological adoption in communication in 1500 CE. For instance, 491 observations (34 countries) in autocracies and 338 observations (26 countries) in democracies are dropped off. The difference in the samples may potentially create an estimation bias.

11. 38 out of 1454 observations are 1 for the DV (autocratization onset).

12. To our knowledge, relevant data for pro-autocratic mobilization in both democratic and autocratic states is unavailable.

13. Due to the limited number of autocracies in the data, we perform the analysis only in democracies.
