# Supplementary material for: Disinformation and Regime Survival
Source: Polit Res Q. 2024 May 27;77(3):1010–25. doi: 10.1177/10659129241252811 (PMC11305955; doi:10.1177/10659129241252811)
Supplement: Supplemental Material - Disinformation and Regime Survival [file sj-pdf-1-prq-10.1177_10659129241252811.pdf]

Appendix for *Disinformation and Regime Survival*

Contents

A Descriptive Statistics and Information about Data ii

B Robustness Check for Democratization and Autocratization Onsets vi

C Robustness Check for Mechanisms xx

A Descriptive Statistics and Information about Data

Table A1. Descriptive Statistics

Variable	Obs.	Max.	Min.	Mean	SD.
Autocracies					
Democratization onset*	1,314	1	0	0.03	0.17
Mobilization for democracy	1,465	4.43	-3.22	0.03	1.51
Mobilization for autocracy	1,465	3.58	-2.34	0.11	1.28
Political polarization	1,465	4.18	-2.48	0.35	1.25
Disinformation	1,466	3.60	-1.60	0.81	0.90
Internet penetration	1,466	1.00	0.00	0.26	0.29
Indoctrination potential in education	1,466	0.90	0.01	0.61	0.20
GDP growth	1,466	55.59	-38.56	2.36	5.74
GDP per capita (log)	1,466	11.49	4.70	7.69	1.45
Population (log)	1,466	21.07	11.30	16.40	1.61
Internet filtering capacity	1,466	2.94	-2.93	0.33	1.23
Democratic stock	1,466	0.40	0.00	0.12	0.06
Regional democracy levels	1,466	0.65	0.24	0.41	0.10
Democracies					
Autocratization onset*	1,454	1	0	0.03	0.16
Mobilization for democracy	1,637	3.87	-2.73	-0.19	1.22
Mobilization for autocracy	1,646	2.53	-2.53	-1.10	1.06
Political polarization	1,656	3.48	-3.87	-0.47	1.34
Disinformation	1,656	2.69	-2.88	-1.03	1.08
Internet penetration	1,656	1.00	0.00	0.48	0.32
Indoctrination potential in education	1,656	0.89	0.02	0.39	0.20
GDP growth	1,656	43.76	-34.78	2.16	4.13
GDP per capita (log)	1,656	11.80	5.26	9.05	1.50
Population (log)	1,656	21.01	11.41	16.12	1.64
Internet filtering capacity	1,656	2.41	-3.06	-0.28	1.02
Democratic stock	1,656	0.73	0.03	0.35	0.18
Regional democracy levels	1,656	0.88	0.24	0.63	0.19

*Observations in the ongoing events are dropped from the sample in the models.

Definition of Democratization and Autocratization Episodes

Democratization or autocratization episodes are defined as periods of substantial and sustained improvements or declines of democratic attributes measured with V-Dem's Electoral Democracy Index (Maerz et al., 2023). According to Maerz et al. (2023), democratization or autocratization episodes begin with an initial annual change of at least +/–0.01 (start inclusion) and must experience an overall change of at least +/–0.10 throughout the episode (cumulative inclusion).

As this threshold can be potentially arbitrary, we also show the robustness of the results using a lower (+/-0.075) and higher (+/-1.250) threshold (Table B4).

Then, we call the first year of episodes of regime transition as democratization or autocratization "onsets." Episodes are considered ongoing as long as the EDI score (i) has an annual change of at least +/–0.01 in one out of every five consecutive years (tolerance), (ii) does not have a reverse annual change of 0.03 or greater (annual turn), and (iii) does not experience a cumulative reverse change of 0.10 over five years (cumulative turn).

The final year of episodes is coded as the year the case experienced a change of at least +/- 0.01 after episode onset and immediately before experiencing one of the three conditions above for termination. Thus, episodes terminate due to prolonged (5-year) periods of stasis, any substantial one-year (0.03) or five-year (0.10) improvement, or a transition to electoral democracy (from electoral autocracy) or autocracy (from electoral democracy)(Maerz et al., 2023).

The ERT dataset also records the outcome of the episodes. For instance, *democratic transition* is observed when an autocratic regime sees sufficient reforms to cross a minimal threshold of democracy (a change from autocracy to democracy) and then holds a founding democratic election. Episodes of democratization that do not result in democratic transition can take one of three paths: a preempted democratic transition, stabilized electoral autocracy, and reverted liberalization. *Democratic breakdown* is coded when a democratic regime regresses to below the minimum threshold of democracy, and one of the following conditions holds: (a) it is considered to be a closed autocracy; (b) it holds a founding authoritarian election for the executive, legislature, or a constituent assembly; or (c) it remains autocratic for a sufficient period to no longer be considered a democracy. Episodes of democratic regression that do not break down can take one of three paths: a preempted democratic breakdown, diminished democracy, and averted regression. We use this information about the episode outcomes in the second stage of two-step models (Table B3).

Table A2 summarizes democratization and autocratization episodes included in the analysis.

Table A2. List of the Autocratization and Democratization Episodes Included in the Models

	Democratization Episodes				Autocratization Episodes			
	Country	Start	End	Outcome	Country	Start	End	Outcome
1	Bahrain	2000	2005	Reverted liberalization	Fiji	2000	2001	Democratic breakdown
2	Burkina Faso	2000	2013	Democratic transition	India	2000	2022	Democratic breakdown
3	Lebanon	2000	2013	Reverted liberalization	Bulgaria	2001	2018	Averted regression
4	Niger	2000	2005	Democratic transition	Philippines	2001	2005	Democratic breakdown
5	Afghanistan	2001	2006	Stabilized electoral autocracy	Bangladesh	2002	2007	Democratic breakdown
6	Ivory Coast	2001	2001	Stabilized electoral autocracy	North Macedonia	2005	2012	Democratic breakdown
7	Burundi	2002	2006	Reverted liberalization	Sri Lanka	2005	2006	Democratic breakdown
8	Fiji	2002	2003	Preempted democratic transition	Thailand	2005	2007	Democratic breakdown
9	Lesotho	2002	2003	Democratic transition	Turkey	2005	2017	Democratic breakdown
10	North Macedonia	2002	2004	Democratic transition	Bolivia	2006	2020	Democratic breakdown
11	Pakistan	2002	2010	Stabilized electoral autocracy	Hungary	2006	2022	Democratic breakdown
12	Iraq	2003	2007	Stabilized electoral autocracy	Nicaragua	2006	2022	Democratic breakdown
13	Seychelles	2003	2007	Stabilized electoral autocracy	Ecuador	2007	2013	Averted regression
14	Georgia	2004	2004	Democratic transition	Mali	2007	2013	Democratic breakdown
15	Rwanda	2004	2009	Stabilized electoral autocracy	Papua New Guinea	2007	2013	Democratic breakdown
16	Central African Republic	2005	2006	Stabilized electoral autocracy	South Korea	2008	2014	Averted regression
17	Kyrgyzstan	2005	2018	Reverted liberalization	Indonesia	2009	2022	Ongoing episodes
18	Liberia	2005	2010	Democratic transition	Niger	2009	2010	Democratic breakdown
19	Maldives	2005	2009	Democratic transition	Serbia	2010	2022	Democratic breakdown
20	Togo	2005	2014	Democratic transition	Ukraine	2010	2014	Democratic breakdown
21	Ukraine	2005	2007	Democratic transition	Zambia	2010	2017	Democratic breakdown
22	Haiti	2006	2012	Stabilized electoral autocracy	Slovenia	2011	2021	Averted regression
23	Nepal	2006	2009	Preempted democratic transition	Maldives	2012	2016	Democratic breakdown
24	Bhutan	2007	2009	Democratic transition	Croatia	2013	2022	Ongoing episodes
25	Angola	2008	2011	Stabilized electoral autocracy	Moldova	2013	2017	Averted regression
26	Thailand	2008	2012	Preempted democratic transition	Burkina Faso	2014	2015	Democratic breakdown
27	Bangladesh	2009	2010	Reverted liberalization	Mauritius	2014	2022	Ongoing episodes

Continued on next page

Table A2 – *Continued from previous page*

28	Malawi	2009	2013	Democratic transition	Tunisia	2014	2022	Democratic breakdown
29	Moldova	2009	2011	Democratic transition	Botswana	2015	2022	Ongoing episodes
30	Armenia	2010	2019	Democratic transition	Lesotho	2015	2017	Averted regression
31	Burma/Myanmar	2010	2019	Reverted liberalization	Mongolia	2015	2022	Ongoing episodes
32	Guinea	2010	2014	Stabilized electoral autocracy	Uruguay	2015	2022	Ongoing episodes
33	Kenya	2010	2014	Preempted democratic transition	Brazil	2016	2022	Ongoing episodes
34	Nigeria	2010	2015	Democratic transition	Niger	2016	2022	Ongoing episodes
35	Sri Lanka	2010	2018	Democratic transition	Philippines	2016	2022	Democratic breakdown
36	Niger	2011	2012	Democratic transition	Poland	2016	2022	Ongoing episodes
37	Tunisia	2011	2012	Democratic transition	United States of America	2016	2022	Ongoing episodes
38	Georgia	2012	2016	Democratic transition	Mali	2017	2022	Democratic breakdown
39	Ivory Coast	2012	2017	Democratic transition	Benin	2018	2020	Democratic breakdown
40	Seychelles	2013	2022	Democratic transition	Burkina Faso	2018	2022	Ongoing episodes
41	Fiji	2014	2022	Ongoing episodes	Guatemala	2018	2022	Ongoing episodes
42	Guinea-Bissau	2014	2019	Democratic transition	Ghana	2019	2022	Ongoing episodes
43	Mali	2014	2014	Democratic transition	Guyana	2019	2022	Ongoing episodes
44	Nepal	2014	2016	Democratic transition	Armenia	2020	2022	Ongoing episodes
45	Burkina Faso	2016	2016	Democratic transition	Ivory Coast	2020	2022	Democratic breakdown
46	Central African Republic	2016	2016	Reverted liberalization	Romania	2021	2022	Ongoing episodes
47	North Macedonia	2017	2019	Democratic transition				
48	The Gambia	2017	2022	Democratic transition				
49	Ethiopia	2018	2019	Reverted liberalization				
50	Malaysia	2018	2019	Reverted liberalization				
51	Maldives	2018	2022	Democratic transition				
52	Ukraine	2019	2020	Preempted democratic transition				
53	Malawi	2020	2022	Democratic transition				
54	Bolivia	2021	2022	Ongoing episodes				
55	Zambia	2021	2022	Ongoing episodes				

Notes: The list only includes episodes that have the potential of regime transformation (either democratic transition or breakdown). Ongoing events are not considered in the second stage of two-step model (Table B3).

B Robustness Check for Democratization and Autocratization Onsets

Table B1. Comparison of Marginal Effects of Disinformation Variable on Regime Transformation using Probit and Linear Probability Models with Fixed Effects

Dependent variable:	Democratization Onset						Autocratization Onset					
	(1) Lpm FE/All		(2) Lpm FE/Lim.		(3) Probit FE		(4) Lpm FE/All		(5) Lpm FE/Lim.		(6) Probit FE	
	β	SE	β	SE	β	SE	β	SE	β	SE	β	SE
Disinformation	−0.039	0.018	−0.119	0.042	−0.735	0.126	0.075	0.031	0.183	0.070	1.206	0.198
AME					−0.123	0.046					0.162	0.044
Sample	Autocracies						Democracies					
Number of Observations	1,311		442		442		1,452		419		419	

Notes: AME is the average marginal effect from the probit model with fixed effects dummies. The second model drops the units which do not have any variation in dependent variables (democratization or autocratization onsets), resulting in the same sample as the probit specification in the third model.

Table B2. Two-Way Fixed-Effects Model without Covariates

	Dependent variable:			
	Dem. Onset		Aut. Onset	
	(7)	(8)	(9)	(10)
**Disinformation**	-0.469^{***}	-0.022^{**}	0.667^{***}	0.059^{**}
	(0.085)	(0.011)	(0.096)	(0.024)
Constant	-0.949^{***}	0.091^{***}	-3.033^{***}	-0.064^{***}
	(0.129)	(0.028)	(0.065)	(0.016)
Sample	Autocracies		Democracies	
Observations	1,711	1,711	1,613	1,613
Log Likelihood	-178.266		-116.062	
Adjusted R^2		0.028		0.178
Country FE	YES	YES	YES	YES
Year FE	YES	YES	YES	YES
Model	Probit	OLS	Probit	OLS

Notes: Standard errors clustered by country. ***, **, * significant at .01, .05, .10, respectively. Countries that do not have multiple-year observations are excluded from the analysis for models.

Table B3. Two-Step Model: The Effect of Disinformation on Regime Change

	Dependent variable:			
	Democratization		Autocratization	
	[I]	Transition	[I]	Breakdown
	(11)	(12)	(13)	(14)
**Disinformation**	**−0.560*****	**−0.368****	**0.496*****	**0.254***
	**(0.121)**	**(0.148)**	**(0.152)**	**(0.184)**
Internet penetration	0.253	−1.196	0.722	−1.572
	(0.750)	(1.075)	(0.937)	(1.366)
Indoctrinate potential	−0.729	0.344	0.948	1.150
	(0.481)	(0.621)	(0.675)	(0.789)
GDP growth	−0.001	0.006	0.008	−0.006
	(0.006)	(0.015)	(0.012)	(0.033)
GDP per capita (log)	−0.179	−0.075	−0.442**	−0.201
	(0.117)	(0.139)	(0.198)	(0.302)
Population (log)	−0.108*	−0.017	0.142	−0.304
	(0.061)	(0.070)	(0.083)	(0.187)
Internet filtering capacity	0.012	0.050	0.341***	0.193
	(0.086)	(0.108)	(0.102)	(0.175)
Democratic stock	3.120**	0.555	3.737**	−4.445
	(1.477)	(1.856)	(1.799)	(2.723)
Episode duration		0.044		0.235***
		(0.053)		(0.076)
Episode duration^2		−0.000		−0.007*
		(0.002)		(0.004)
Constant	2.227*	−0.793	−0.323	3.062
	(1.235)	(1.528)	(2.052)	(3.383)
ρ	-	0.000	-	0.000
Sample	Autocracies		Democracies	
Region FE	✓		✓	
Nonlinear time trend	✓		✓	
Log Likelihood	-1416.110		-635.928	
Total obs.	1,622		1,768	
Censored obs.	1,277		1,425	
Obs. in outcome stage	345		343	

Notes: Standard errors clustered by country. ***, **, * significant at .01, .05, .10, respectively.

Table B4. Identifying Regime Transformation Episodes with Lower/Higher Thresholds

	Dependent variable:			
	Lower threshold (0.075)		*Higher threshold (1.250)*	
	Dem. Onset	Aut. Onset	Dem. Onset	Aut. Onset
	(15)	(16)	(17)	(18)
**Disinformation**	-0.311^{***}	0.206^{***}	-0.416^{***}	0.387^{***}
	(0.062)	(0.079)	(0.065)	(0.085)
Internet Penetration	-0.593	0.413	-0.944^{*}	0.500
	(0.447)	(0.556)	(0.534)	(0.632)
Indoctrination potential	-0.577^{**}	0.258	-0.297	0.786
	(0.251)	(0.364)	(0.239)	(0.515)
GDP growth	-0.032^{***}	-0.012	-0.036^{***}	-0.028^{***}
	(0.010)	(0.010)	(0.010)	(0.011)
GDP per capita (log)	-0.074	-0.153	-0.039	-0.227^{**}
	(0.069)	(0.093)	(0.079)	(0.109)
Population (log)	-0.062^{*}	0.094^{**}	-0.070^{*}	0.032
	(0.037)	(0.045)	(0.038)	(0.054)
Internet filtering capacity	0.014	0.246^{***}	0.094	0.344^{***}
	(0.054)	(0.062)	(0.058)	(0.073)
Democratic stock	-0.018	0.572	-0.577	1.301
	(0.941)	(0.848)	(1.082)	(1.005)
Constant	-0.010	-2.311^{**}	-0.079	-1.434
	(0.818)	(0.980)	(0.966)	(1.016)
Sample	Aut.	Dem.	Aut.	Dem.
Regional FE	✓	✓	✓	✓
Nonlinear time trend	✓	✓	✓	✓
Observations	1,313	1,431	1,329	1,503
Log-Likelihood	-159.177	-162.418	-119.251	-108.763

Notes: Standard errors clustered by country. ***, **, * significant at .01, .05, .10, respectively.

Table B5. Duration Model (Beck, Katz, and Tucker, 1998)

	Dependent variable:	
	Dem. Onset	Aut. Onset
	(19)	(20)
**Disinformation**	-1.021^{***}	0.048
	(0.284)	(0.302)
Internet Penetration	-3.658^{**}	3.672^{**}
	(1.601)	(1.772)
Indoctrination potential	-2.435^{***}	0.468
	(0.813)	(1.695)
GDP growth	-0.081^{***}	-0.035
	(0.023)	(0.033)
GDP per capita (log)	-0.014	-1.248^{***}
	(0.245)	(0.400)
Population (log)	-0.161	0.198
	(0.108)	(0.158)
Internet filtering capacity	-0.056	0.864^{***}
	(0.190)	(0.295)
Democratic stock	14.388^{***}	4.977
	(3.095)	(3.216)
t	-4.850^{***}	-1.396^{***}
	(0.538)	(0.126)
t^2	0.121^{***}	0.037^{***}
	(0.013)	(0.003)
t^3	-0.001^{***}	-0.000^{***}
	(0.000)	(0.000)
Constant	0.691	2.289
	(2.730)	(4.431)
Sample	Aut.	Dem.
Regional FE	✓	✓
Nonlinear time trend	✓	✓
Observations	1,314	1,454
Log-likelihood	-60.038	-99.648

Notes: Standard errors clustered by country. ***, **, * significant at .01, .05, .10, respectively.

Table B6. Residual as DV (McGrath, 2015)

	Dependent variable:	
	Dem. Onset	Aut. Onset
	(21)	(22)
**Disinformation**	0.777***	−0.674***
	(0.214)	(0.252)
Internet Penetration	1.398	−1.209
	(1.724)	(1.799)
Indoctrination potential	0.792	−0.496
	(0.845)	(1.363)
GDP growth	0.066**	0.042
	(0.030)	(0.032)
GDP per capita (log)	0.125	0.532
	(0.242)	(0.422)
Population (log)	0.134	−0.227
	(0.115)	(0.193)
Internet filtering capacity	−0.098	−0.736***
	(0.181)	(0.224)
Democratic stock	0.619	−4.069
	(3.596)	(4.001)
Constant	−0.117	3.619
	(2.691)	(3.231)
Sample	Aut.	Dem.
Regional FE	✓	✓
Nonlinear time trend	✓	✓
Observations	1,314	1,454
Log-likelihood	-149.593	-142.506

Notes: Standard errors clustered by country. ***, **, * significant at .01, .05, .10, respectively.

Table B7. Different Lags of the Disinformation Variable

	(23)	(24)	(25)	(26)	(27)	(28)	(29)	(30)	(31)	(32)	(33)	(34)
Dependent variable	Democratization Onset						Autocratization Onset					
Lag of disinformation variable	t	t-1	t-2	t-3	t-4	t-5	t	t-1	t-2	t-3	t-4	t-5
**Disinformation**	-0.322^{***}	-0.169^{***}	-0.151^{***}	-0.148^{**}	-0.170^{***}	-0.106	0.285^{***}	0.066	0.014	0.065	0.117	0.146^{**}
	(0.065)	(0.055)	(0.054)	(0.060)	(0.061)	(0.067)	(0.077)	(0.072)	(0.074)	(0.078)	(0.075)	(0.070)
Sample	Autocracies						Democracies					
Regional FE	✓	✓	✓	✓	✓	✓	✓	✓	✓	✓	✓	✓
Nonlinear time trend	✓	✓	✓	✓	✓	✓	✓	✓	✓	✓	✓	✓
Observations	1,314	1,255	1,192	1,130	1,073	1,016	1,454	1,387	1,320	1,254	1,073	1,117
Log-Likelihood	−150.368	−144.167	−138.953	−129.026	−119.980	−114.188	−143.300	−142.495	−135.513	−134.783	−119.980	−131.525

Notes: The model specification is the same as the Model 1 of Table 1. Control variables are also included. Standard errors clustered by country. ***, **, * significant at .01, .05, .10, respectively.

Table B8. Using Change in Disinformation Instead of the Level

	Dependent variable:	
	Dem. Onset	Aut. Onset
	(35)	(36)
**Disinformation**	-1.653^{***}	1.136^{***}
	(0.349)	(0.161)
Internet Penetration	-0.163	-0.392
	(0.450)	(0.533)
Indoctrination potential	-0.291	0.597^{*}
	(0.273)	(0.333)
GDP growth	-0.024^{***}	-0.017
	(0.009)	(0.011)
GDP per capita (log)	0.036	-0.248^{***}
	(0.075)	(0.095)
Population (log)	0.004	0.042
	(0.038)	(0.045)
Internet filtering capacity	-0.072	0.285^{***}
	(0.062)	(0.063)
Democratic stock	-0.101	0.714
	(0.853)	(0.661)
Constant	-2.032^{**}	-0.979
	(0.949)	(1.003)
Sample	Aut.	Dem.
Regional FE	✓	✓
Nonlinear time trend	✓	✓
Observations	1,255	1,387
Log-Likelihood	-132.754	-129.820

Notes: Change in disinformation variable is a difference between disinformation at t and $t-1$. Standard errors clustered by country. ***, **, * significant at .01, .05, .10, respectively.

Table B9. Including State Capacity as a Control

	Dependent variable:	
	Dem. Onset	Aut. Onset
	(37)	(38)
**Disinformation**	-0.356^{***}	0.348^{***}
	(0.073)	(0.085)
State capacity	0.148	0.976^{***}
	(0.100)	(0.224)
Internet Penetration	-1.370^{**}	0.682
	(0.614)	(0.567)
Indoctrination potential	-0.544^{*}	1.453^{***}
	(0.302)	(0.390)
GDP growth	-0.037^{***}	-0.024^{**}
	(0.010)	(0.012)
GDP per capita (log)	−0.073	-0.673^{***}
	(0.083)	(0.146)
Population (log)	−0.069	−0.028
	(0.044)	(0.053)
Internet filtering capacity	0.076	0.199^{***}
	(0.062)	(0.056)
Democratic stock	−0.907	2.000^{**}
	(1.302)	(0.830)
Constant	−0.000	2.481^{**}
	(1.078)	(1.070)
Sample	Aut.	Dem.
Regional FE	✓	✓
Nonlinear time trend	✓	✓
Observations	900	1,019
Log-Likelihood	-113.043	-88.860

Notes: Standard errors clustered by country. ***, **, * significant at .01, .05, .10, respectively.

Table B10. Including State Repression Capacity as a Control

	Dependent variable:	
	Dem. Onset	Aut. Onset
	(39)	(40)
**Disinformation**	-0.400^{***}	0.332^{***}
	(0.092)	(0.084)
Repression capacity	-0.351^{***}	-0.046
	(0.121)	(0.132)
Internet penetration	-0.929	1.184^{*}
	(0.658)	(0.623)
Indoctrination potential	-0.250	1.680^{***}
	(0.321)	(0.383)
GDP growth	-0.031^{***}	-0.001
	(0.011)	(0.010)
GDP per capita (log)	0.002	-0.337^{***}
	(0.088)	(0.093)
Population (log)	-0.139^{**}	0.142^{***}
	(0.062)	(0.051)
Internet filtering capacity	0.049	0.111^{**}
	(0.060)	(0.053)
Democratic stock	-2.551^{**}	2.244^{**}
	(0.998)	(0.934)
Constant	1.221	-2.290^{*}
	(1.465)	(1.236)
Sample	Aut.	Dem.
Regional FE	✓	✓
Nonlinear time trend	✓	✓
Observations	676	793
Log-Likelihood	-95.498	-56.262

Notes: Standard errors clustered by country. ***, **, * significant at .01, .05, .10, respectively. Among 4 types of repressions (imprisonment, torturer, extrajudicial killing, and disappearance) introduced by Fariss and Schnakenberg (2014), we use extrajudicial killing as a representative repertoire of repression. However, we report that the results are similar by controlling for other types of repressions.

Table B11. Including Government Control over Social Media as a Control

	Dependent variable:	
	Dem. Onset	Aut. Onset
	(41)	(42)
**Disinformation**	-0.356^{***}	0.264^{***}
	(0.061)	(0.085)
Social media control	-0.100^{**}	-0.112
	(0.045)	(0.082)
Internet penetration	-0.621	0.438
	(0.460)	(0.537)
Indoctrination potential	-0.455^{*}	0.318
	(0.236)	(0.414)
GDP growth	-0.030^{***}	-0.019^{*}
	(0.010)	(0.010)
GDP per capita (log)	-0.035	-0.218^{**}
	(0.072)	(0.099)
Population (log)	-0.062^{*}	0.077
	(0.037)	(0.049)
Internet filtering capacity	0.005	0.296^{***}
	(0.054)	(0.064)
Democratic stock	0.643	1.709^{*}
	(0.968)	(0.982)
Constant	-0.202	-1.458
	(0.867)	(0.984)
Sample	Aut.	Dem.
Regional FE	✓	✓
Nonlinear time trend	✓	✓
Observations	1,314	1,454
Log-Likelihood	-149.749	-143.030

Notes: Standard errors clustered by country. ***, **, * significant at .01, .05, .10, respectively.

Table B12. Including Judicial and Legislative Constraints as Controls

	Dependent variable:	
	Dem. Onset	Aut. Onset
	(43)	(44)
**Disinformation**	-0.326^{***}	0.279^{***}
	(0.067)	(0.082)
Judicial constraints on executive	-0.278	-0.157
	(0.385)	(0.556)
Legislative constraints on executive	1.004^{***}	0.090
	(0.298)	(0.481)
Internet penetration	-0.448	0.399
	(0.428)	(0.530)
Indoctrination potential	-0.284	0.247
	(0.257)	(0.362)
GDP growth	-0.028^{***}	-0.018^{*}
	(0.010)	(0.010)
GDP per capita (log)	-0.082	-0.222^{**}
	(0.071)	(0.103)
Population (log)	-0.082^{*}	0.072
	(0.042)	(0.048)
Internet filtering capacity	0.087	0.302^{***}
	(0.067)	(0.066)
Democratic stock	-1.217	1.528^{*}
	(1.332)	(0.894)
year_2000	0.084^{**}	0.120^{***}
	(0.037)	(0.039)
I(year_2000^2)	-0.003^{**}	-0.005^{***}
	(0.002)	(0.002)
Constant	0.079	-1.384
	(0.953)	(0.998)
Sample	Aut.	Dem.
Regional FE	✓	✓
Nonlinear time trend	✓	✓
Observations	1,301	1,454
Log-Likelihood	-141.994	-143.393

Notes: Standard errors clustered by country. ***, **, * significant at .01, .05, .10, respectively.

Table B13. Including Coup as a Control

	Dependent variable:	
	Dem. Onset	Aut. Onset
	(45)	(46)
**Disinformation**	-0.326^{***}	0.261^{***}
	(0.063)	(0.075)
Coup	-0.175	1.962^{***}
	(0.267)	(0.413)
Internet penetration	-0.590	0.494
	(0.469)	(0.530)
Indoctrination potential	-0.480^{*}	0.555
	(0.257)	(0.381)
GDP growth	-0.028^{***}	-0.016
	(0.010)	(0.011)
GDP per capita (log)	-0.053	-0.275^{***}
	(0.072)	(0.100)
Population (log)	-0.065^{*}	0.105^{**}
	(0.036)	(0.049)
Internet filtering capacity	0.031	0.294^{***}
	(0.053)	(0.063)
Democratic stock	-0.025	2.173^{**}
	(0.978)	(0.907)
Constant	-0.036	-1.818^{*}
	(0.842)	(0.998)
Sample	Aut.	Dem.
Regional FE	✓	✓
Nonlinear time trend	✓	✓
Observations	1,314	1,454
Log-Likelihood	-150.202	-139.254

Notes: Standard errors clustered by country. ***, **, * significant at .01, .05, .10, respectively.

Table B14. Excluding the Commonly Studied Cases (Russia, China, and the United States)

	Dependent variable:	
	Dem. Onset	Aut. Onset
	(47)	(48)
**Disinformation**	-0.318^{***}	0.272^{***}
	(0.065)	(0.078)
Internet penetration	-0.577	0.467
	(0.470)	(0.575)
Indoctrination potential	-0.493^{*}	0.425
	(0.255)	(0.398)
GDP growth	-0.028^{***}	-0.019^{*}
	(0.010)	(0.010)
GDP per capita (log)	-0.037	-0.256^{***}
	(0.074)	(0.099)
Population (log)	-0.055	0.042
	(0.039)	(0.048)
Internet filtering capacity	0.026	0.304^{***}
	(0.056)	(0.064)
Democratic stock	-0.097	1.700^{*}
	(0.995)	(0.963)
Constant	-0.262	-0.815
	(0.883)	(0.939)
Sample	Aut.	Dem.
Regional FE	✓	✓
Nonlinear time trend	✓	✓
Observations	1,268	1,437
Log-Likelihood	-149.948	-138.007

Notes: Standard errors clustered by country. ***, **, * significant at .01, .05, .10, respectively.

C Robustness Check for Mechanisms

Table C1. Instrument Variable Approach (Mechanism)

	Dependent variable:					
	Dem. Mobilization		Aut. Mobilization		Polarization	
	(1)	(2)	(3)	(4)	(5)	(6)
First-stage regression. DV: Internet penetration						
Comm.tech. in 1500 CE	−0.324**	0.445***	−0.324**	0.445***	−0.324**	0.445***
	(0.142)	(0.086)	(0.142)	(0.086)	(0.142)	(0.086)
Ave. Internet penetration	1.060***	0.739***	1.060***	0.739***	1.060***	0.739***
	(0.065)	(0.055)	(0.065)	(0.055)	(0.065)	(0.055)
Comm.tech. in 1500 CE*Ave. Internet penetration	0.151**	−0.282***	0.151**	−0.282***	0.151**	−0.282***
	(0.065)	(0.030)	(0.065)	(0.030)	(0.065)	(0.030)
F statistics	5.596**	86.511***	5.596**	86.511***	5.596**	86.511***
Second-stage regression. DV: Mobilization/Polarization						
**Disinformation**	−0.163	0.302***	0.109	0.345**	0.050	0.417***
	(0.277)	(0.112)	(0.235)	(0.143)	(0.238)	(0.078)
Internet Penetration (predicted)	12.561	−1.307	4.016	0.027	12.374	−0.867
	(8.643)	(1.295)	(6.841)	(1.456)	(7.929)	(1.281)
Indoctrination potential	1.260	2.040*	0.986	0.934	2.233	1.809**
	(2.105)	(1.057)	(1.442)	(0.899)	(1.635)	(0.822)
GDP growth	−0.003	0.002	−0.006	0.004	0.008	−0.003
	(0.012)	(0.006)	(0.008)	(0.007)	(0.009)	(0.005)
GDP per capita (log)	−0.506***	0.025	0.118	−0.047	−0.323***	−0.166
	(0.191)	(0.144)	(0.112)	(0.107)	(0.080)	(0.106)
Population (log)	5.669	−1.657***	1.065	−0.309	4.969	−0.725
	(3.552)	(0.625)	(2.887)	(0.674)	(3.409)	(0.633)
Internet filtering capacity	0.238	−0.035	0.189	−0.149	0.402**	−0.161*
	(0.224)	(0.084)	(0.172)	(0.110)	(0.186)	(0.096)
Democratic stock	40.484	4.066	28.782	2.543	45.710	6.279
	(32.592)	(4.114)	(23.809)	(5.082)	(30.053)	(5.707)
Regional democracy levels	6.405	−0.759	0.342	0.762	6.689	−3.650
	(7.699)	(2.911)	(5.526)	(2.969)	(6.005)	(3.126)
Constant	−97.862	28.614**	−20.654	3.678	−87.435	15.088
	(64.482)	(11.389)	(51.877)	(12.031)	(61.384)	(11.224)
Country FE	✓	✓	✓	✓	✓	✓
Year FE	✓	✓	✓	✓	✓	✓
Sample	Aut.	Dem.	Aut.	Dem.	Aut.	Dem.
Observations	901	1,256	901	1,256	901	1,256
Adjusted R^2	0.328	0.852	0.771	0.884	0.282	0.933

Notes: Standard errors clustered by country. ***, **, * significant at .01, .05, .10, respectively. Countries that do not have multiple-year observations are excluded from the analysis.

Table C2. Alternative Data (Mechanism)

	Dependent variable:		
	Dem. Mobilization		Polarization
Sample	Aut.	Dem.	Dem.
	(7)	(8)	(9)
**Disinformation**	0.285	0.384***	0.098**
	(0.198)	(0.143)	(0.048)
Internet penetration	−0.869	−1.221	−0.226
	(0.532)	(0.781)	(0.333)
Indoctrination potential	−1.140**	0.961	−0.811
	(0.581)	(1.210)	(0.520)
GDP growth	−0.020	−0.032**	−0.002
	(0.012)	(0.016)	(0.011)
GDP per capita (log)	−0.168	−0.581**	0.122
	(0.196)	(0.251)	(0.165)
Population (log)	1.088	−1.648	−1.643**
	(0.728)	(1.269)	(0.818)
Internet filtering capacity	−0.223	−0.036	0.051
	(0.152)	(0.253)	(0.185)
Democratic stock	−7.893	−6.796	−15.907***
	(8.094)	(9.167)	(3.957)
Regional democracy levels	−0.831	5.697*	2.802
	(2.527)	(2.957)	(4.160)
Constant	−16.838	34.004	33.874**
	(13.016)	(22.607)	(13.750)
Country FE	✓	✓	✓
Year FE	✓	✓	✓
Model	NB	NB	OLS
Observations	1,311	1,396	144
Adjusted R^2			0.711
Log-Likelihood	-2101.413	-2361.947	

Notes: Standard errors clustered by country. ***, **, * significant at .01, .05, .10, respectively. Models 49-50 use the negative binomial regression model as the DV is the event counts for pro-democratic mobilization.
